# Pharmacokinetics of eribulin mesylate in cancer patients with normal and impaired renal function

**DOI:** 10.1007/s00280-015-2878-5

**Published:** 2015-10-03

**Authors:** Antoinette R. Tan, John Sarantopoulos, Lucy Lee, Larisa Reyderman, Yi He, Martin Olivo, Sanjay Goel

**Affiliations:** Rutgers Cancer Institute of New Jersey, New Brunswick, NJ 08903 USA; Department of Medical Oncology, Levine Cancer Institute, Carolinas HealthCare System, Charlotte, NC 28204 USA; Institute for Drug Development, Cancer Therapy and Research Center, University of Texas Health Science Center San Antonio, San Antonio, TX 78229 USA; Department of Clinical Pharmacology and Translational Medicine, Eisai Inc., Woodcliff Lake, NJ 07677 USA; Department of Clinical Pharmacology and Translational Medicine, Oncology, Eisai Inc., Woodcliff Lake, NJ 07677 USA; Department of Biostatistics, Oncology, Eisai Inc., Woodcliff Lake, NJ 07677 USA; Department of Oncology Clinical Development, Eisai Inc., Woodcliff Lake, NJ 07677 USA; Department of Medical Oncology, Montefiore Medical Center, Albert Einstein College of Medicine, 1695 Eastchester Road, Bronx, NY 10461 USA

**Keywords:** Renal function, Renal impairment, Cancer patients, Eribulin, Pharmacokinetics

## Abstract

**Purpose:**

To evaluate the effect of renal impairment on eribulin mesylate pharmacokinetics following a single dose in adults with advanced solid tumors.

**Methods:**

Patients were grouped by renal function: moderate impairment (creatinine clearance [CrCl] 30–50 mL/min), severe impairment (CrCl 15–29 mL/min), or normal (CrCl ≥80 mL/min). During each 21-day cycle, eribulin mesylate doses (days 1 and 8) were administered intravenously: moderate, 1.1 mg/m^2^ (except cycle 1 day 1, 1.4 mg/m^2^); severe, 0.7 mg/m^2^; normal, 1.4 mg/m^2^.

**Results:**

Nineteen patients were enrolled (normal, *n* = 6; moderate, *n* = 7; severe, *n* = 6). Renal impairment was associated with an increased mean dose-normalized area under the concentration–time curve (ratios for moderate/normal and severe/normal: 1.49; 90 % confidence interval [CI] 0.9, 2.45). CrCl and renal function correlated positively, with a numerically small slope (0.0184; 90 % CI −0.00254, 0.0394). A simulated dose reduction to eribulin 1.1 mg/m^2^ in patients with moderate or severe renal impairment achieved the same exposure as 1.4 mg/m^2^ in those with normal renal function. All groups had similar toxicity profiles, with no unexpected adverse events.

**Conclusions:**

Renal impairment decreased eribulin clearance and increased exposure. Pharmacokinetic evaluation supports an eribulin dose reduction to 1.1 mg/m^2^ in patients with moderate or severe renal impairment.

**ClinicalTrials.gov Identifier:**

NCT01418677.

## Introduction

Eribulin mesylate (eribulin; also known as eribulin mesylate; Eisai Inc., NJ, USA) is a synthetic analogue of the biologically active macrocyclic portion of halichondrin B, a natural product isolated from the marine sponge *Halichondria okadai* [1]. Halichondrin B is a large polyether macrolide that exerts potent anticancer effects in cell-based and animal models of cancer [1–3]. In the USA, eribulin is indicated for the treatment of patients with metastatic breast cancer who have previously received at least 2 chemotherapeutic regimens for the treatment of metastatic disease. Prior therapy should have included an anthracycline and a taxane in either the adjuvant or metastatic setting. The recommended dose is eribulin mesylate 1.4 mg/m^2^ (equivalent to 1.23 mg/m^2^ eribulin, expressed as free base), administered intravenously over 2–5 min on days 1 and 8 of a 21-day cycle [4, 5].

In humans, eribulin (free base) has a rapid distribution phase followed by a prolonged elimination phase, with a mean terminal half-life (*t*_½_) of approximately 40 h [6, 7]. It has a large volume of distribution at steady state (*V*_ss_ 43–114 L/m^2^) [4, 6] and low plasma total clearance (CL_tot_ 1.16–2.42 L/h/m^2^) [7, 8]. Metabolism accounts for a minor portion of eribulin clearance [9]. Following administration of ^14^C-eribulin mesylate 2 mg to patients, unchanged parent compound accounted for almost all of the eribulin-derived radioactivity [9]. Hepatic impairment affects the disposition of eribulin by decreasing clearance and prolonging elimination half-life, resulting in increased exposure to eribulin [10]. Consequently, the US eribulin package insert recommends that the eribulin mesylate dose be reduced from 1.4 to 1.1 mg/m^2^ in patients with *mild* (Child–Pugh A) hepatic impairment or to 0.7 mg/m^2^ in patients with *moderate* (Child–Pugh B) hepatic impairment [4]. Renal elimination is a minor route for eribulin excretion, with less than 10 % of the drug excreted unchanged in urine; the majority is excreted unchanged in feces [9]. Although it cannot be directly measured, biliary excretion may also contribute substantially to eribulin clearance.

In toxicokinetic studies, no significant accumulation of eribulin was observed with weekly administration (administered once per week for 3 weeks) [11]. Eribulin exposure following the second or third weekly dose of the first cycle is comparable to that achieved following a single dose [4]. Exposure is dose-related at doses of 0.25–4.0 mg/m^2^ [6, 7]. Population pharmacokinetic (PK) analyses showed that eribulin clearance is affected by levels of serum albumin, alkaline phosphatase, and bilirubin [12]. The effects of age, sex, race, and concomitant medications (cytochrome P450 inhibitors and inducers) on clearance were not significant. After normalizing for body weight, creatinine clearance (CrCl) had no effect on eribulin clearance.

Based on the PK characteristics of eribulin, the primary assessment in this study was conducted on eribulin in plasma and followed the principles outlined in the US Food and Drug Administration (FDA) Renal Impairment Study Guidance for Industry [13]. The primary objective was to study the influence of moderate and severe renal impairment on the PK of eribulin following a single intravenous (i.v.) administration of eribulin mesylate to patients with cancer. The secondary objective was to explore the safety and tolerability of eribulin mesylate when administered repeatedly in patients with moderate and severe renal impairment, as well as in those with normal renal function.

## Materials and methods

### Study design

This was a multicenter, open-label, nonrandomized, sequential-cohort trial in patients with advanced or metastatic solid tumors who were no longer responding to available therapy. Patients at 6 centers received eribulin mesylate administered as a single i.v. infusion over 2–5 min on days 1 and 8 of a 21-day cycle. The dose was determined by each patient’s renal function (normal renal function: CrCl ≥80 mL/min; moderate renal impairment: CrCl 30–50 mL/min; severe renal impairment: CrCl 15–29 mL/min). CrCl rates were estimated by the Cockcroft–Gault formula. Patients with normal renal function were matched to those with moderate or severe renal impairment with regard to sex, age, height, and weight, to the maximum extent possible. To assure selection of a suitable eribulin mesylate dose for patients with renal impairment, the study initially recruited and dosed only those with moderate renal impairment, who received eribulin mesylate 1.4 mg/m^2^ on cycle 1 day 1, and then 1.1 mg/m^2^ on cycle 1 day 8 and for all subsequent doses. Patients with severe renal impairment received eribulin mesylate 0.7 mg/m^2^, and those with normal renal function received 1.4 mg/m^2^ on days 1 and 8 of each 21-day cycle. Patients continued to receive the study drug on days 1 and 8 of each cycle until their study participation ended. To evaluate the need for dose adjustment for patients with severe renal impairment, eribulin exposure was compared with predicted exposure based on a population PK model. Institutional review board approvals were obtained from all clinical sites prior to study initiation.

### Ethical approval

All procedures performed in this study involving human participants were in accordance with the ethical standards of the institutional and/or national research board and with the principles of the 2008 Declaration of Helsinki.

### Patients

To be eligible to participate in the trial, men and women had to meet the following key inclusion criteria: aged 18 years or older at the time of informed consent; histologically or cytologically confirmed advanced solid tumors that had progressed following standard therapy or for which no standard therapy existed; renal function in 1 of 3 categories: normal (CrCl ≥80 mL/min), moderate impairment (CrCl 30–50 mL/min), or severe impairment (CrCl 15–29 mL/min); resolution of all chemotherapy- or radiation-related toxicities to grade 1 or lower; Eastern Cooperative Oncology Group Performance Status score of 0, 1, or 2; adequate liver function as evidenced by bilirubin levels of up to 1.5× the upper limit of normal (ULN) and levels of alkaline phosphatase, alanine aminotransferase, and aspartate aminotransferase up to 3× ULN; and adequate bone marrow function as evidenced by absolute neutrophil count of at least 1.5 × 10^9^/L, hemoglobin concentration of at least 10.0 g/dL and platelet count of at least 100 × 10^9^/L. All patients provided written informed consent and were willing and able to comply with all aspects of the protocol. Exclusion criteria included: hypersensitivity to halichondrin B or its derivatives; neuropathy greater than grade 2; radiation therapy to more than 30 % of bone marrow; organ allografts that required immunosuppression; clinically significant illness requiring medical treatment during the 8 weeks, or a clinically significant infection during the 4 weeks preceding the first dose; corrected QT (QTc) interval longer than 500 ms; positive test result for human immunodeficiency virus, hepatitis A, B, or C; significant cardiovascular impairment such as a history of congestive heart failure greater than grade 2, unstable angina or myocardial infarction within the past 6 months, or serious cardiac arrhythmia; brain or subdural metastases; and presence of meningeal carcinomatosis.

### Pharmacokinetic assessments

Blood samples for eribulin PK analysis were collected on cycle 1 day 1, predose (time 0) and at the following postdose time points: 15 and 30 min, and 1, 2, 4, 6, 10, 24, 48, 72, 96, 120, 144, and 168 h. Additional blood samples were collected 30 min and 24 h postdose to determine the unbound fraction of total plasma drug concentration. Plasma concentrations of eribulin were estimated using noncompartmental analysis to determine: area under the concentration–time curve from time zero extrapolated to infinity (AUC_0–inf_), AUC from time zero to last measurable time point (AUC_0–*t*_), maximum observed concentration (*C*_max_), *t*_½_, CL_tot_, *V*_ss_, and unbound fraction of plasma drug concentration. Plasma concentrations of eribulin were quantified by liquid chromatography with tandem mass spectrometry using a previously validated assay [14]; the lower limit of quantification was 0.2 ng/mL.

### Safety assessments

All adverse events (AEs) and serious AEs (SAEs) were recorded using the Common Terminology Criteria for Adverse Events (CTCAE) v4.0 grades (for both increasing and decreasing severity). Safety assessments also included: monitoring and recording of all concomitant medications; regular monitoring of hematology, blood chemistry, and urine analysis values; periodic assessment of vital signs and electrocardiograms; and physical examinations. All hematology, blood chemistry (including pregnancy test, if applicable), and urinalysis samples were obtained before study-drug administration. Data from the safety assessments were reviewed before dispensing of the study drug at the beginning of each cycle. Treatment-emergent, markedly abnormal laboratory values were defined as an increase from baseline to a postbaseline CTCAE grade 2 or higher (grade 3 or higher for phosphate).

### Efficacy assessments

Patients were evaluated for efficacy by serial computerized tomography scans of the chest, abdomen, and pelvis, every 2 cycles (6 weeks). Best overall response was based on the tumor response evaluation as determined by the investigator, according to Response Evaluation Criteria in Solid Tumors 1.1 [15]. Responses of complete response (CR) and partial response (PR) must have been confirmed at least 4 weeks following the first response.

### Statistical assessments

To compare eribulin exposure in patients with renal impairment with that in patients with normal renal function, exposure (AUC_0–inf_ and *C*_max_ values) was dose-normalized to account for different doses tested in each treatment group. The relationship between the individual eribulin PK parameters and estimated renal function (CrCl) was analyzed using a linear regression model, which was sufficient to describe the data. The log-transformed PK parameters (dose-normalized AUC_0–inf_, AUC_0–*t*_, *C*_max_, and CL_tot_) were the dependent variables, while CrCl was the independent variable. Individual CrCls were estimated by the Cockcroft–Gault formula using serum creatinine data from screening (day −21 to day −1), or from baseline visits if the screening value was missing. Safety variables were analyzed using descriptive summary statistics and changes from baseline were evaluated by renal function group. No formal statistical analysis of the efficacy data was conducted.

## Results

### Patient disposition

Nineteen patients were enrolled and treated with eribulin mesylate (normal renal function, *n* = 6; moderate renal impairment, *n* = 7; severe renal impairment, *n* = 6) between October 2011 and July 2013 (data cut-off). Patient demographic and other baseline characteristics are presented in Table 1. Overall, the majority of patients were white (63.2 %), female (63.2 %), and 70 years of age or older (52.6 %). More than half of the patients in the moderate and severe renal impairment groups were females who were age 69 and older. Based on screening or baseline serum creatinine values, the mean estimated CrCl was 89.6 mL/min (±standard deviation [SD] 9.1) for the normal renal function group, 42.6 mL/min (±SD 6.3) for the moderate renal impairment group, and 24.4 mL/min (±SD 4.9) for the severe renal impairment group. No single tumor type was predominant among the three groups (Table 1).

**Table 1:** Study population baseline characteristics

Parameter	Normal renal function (*n* = 6)	Moderate renal impairment (*n* = 7)	Severe renal impairment (*n* = 6)	Overall (*N* = 19)
Eribulin mesylate dose	1.4 mg/m^2^	1.4 mg/m^2^	0.7 mg/m^2^	
Women, *n*	4	6	2	12
Age, years				
Median	63.5	73.0	73.5	70.0
Range	33–72	34–82	56–81	33–82
Race, *n*				
White	4	6	2	12
Black/African–American	2	1	4	7
Weight, kg				
Mean (SD)	78.2 (20.2)	77.0 (16.4)	67.1 (9.3)	74.3 (15.9)
Range	47.4–97.3	45.9–101.2	54.7–81.2	45.9–101.2
Height, cm				
Mean (SD)	166.7 (12.4)	159.8 (9.5)	170.9 (7.3)	165.4 (10.5)
Range	158.0–191.0	149.4–178.6	163.3–182.0	149.4–191.0
BSA, m^2^				
Mean (SD)	1.860 (0.264)	1.796 (0.192)	1.797 (0.069)	1.816 (0.184)
Range	1.48–2.21	1.45–2.03	1.71–1.90	1.45–2.21
Tumor type				
Breast	2	2	0	4
Cervix uteri	0	1	0	1
Esophagus	1	0	1	2
Mouth	1	0	0	1
Lung/bronchus	2	1	1	4
Ovary	0	2	1	3
Prostate gland	0	0	1	1
Testis	0	1	0	1
Urinary bladder	0	0	2	2
Number of previous anticancer regimens, *n*				
1	1	0	1	2
2	0	1	1	2
3	0	0	1	1
≥4	5	6	3	14
ECOG status, *n*				
0	1	0	2	3
1	5	7	4	16
CrCl, mL/min				
Mean (SD)	89.6 (9.1)	42.6 (6.3)	24.4 (4.9)	51.7 (28.4)
Range	81.4–107.2	29.8–48.1	16.7–29.7	16.7–107.2

*BSA* body surface area, *CrCl* creatinine clearance, *ECOG* Eastern Cooperative Oncology Group, *SD* standard deviation

### Plasma concentration–time profiles and pharmacokinetic parameters

**Fig. 1:**
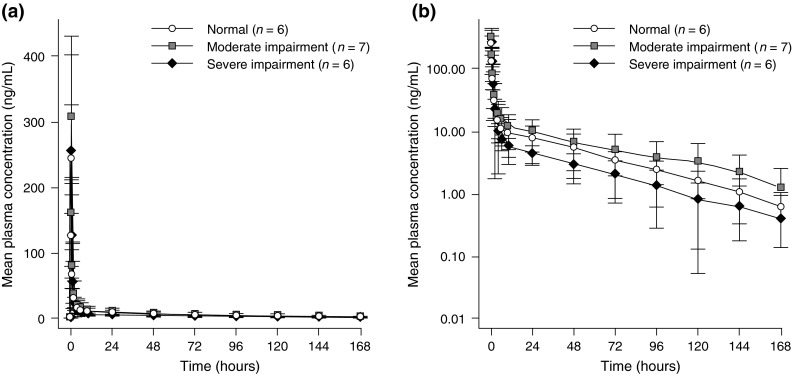
**a** Linear and **b** semi-log plots of mean (±standard deviation) eribulin plasma concentration versus time following a single intravenous administration of eribulin mesylate 1.4 mg/m^2^ to patients with normal renal function, 1.4 mg/m^2^ to patients with moderate renal impairment, and 0.7 mg/m^2^ to patients with severe renal impairment. Data shown as mean ± standard deviation

All 19 enrolled patients had evaluable PK data and were included in the analysis. Figure 1 shows the mean ± SD eribulin plasma concentration–time profiles by renal impairment group. Eribulin exhibited multiphasic disposition following i.v. drug administration. There was a rapid distribution phase followed by a prolonged elimination phase. At the same 1.4 mg/m^2^ dose, patients with moderate renal impairment had higher eribulin plasma concentrations than those with normal renal function. Patients with severe renal impairment received a lower dose (0.7 mg/m^2^) and had a lower eribulin plasma concentration profile than those with normal renal function. The variability in eribulin exposure was comparable across groups; the interpatient variability (%CV) for AUC_0–inf_ was 49, 59, and 39 % for patients with normal renal function, moderate renal impairment, or severe renal impairment, respectively.

Table 2 shows eribulin PK parameters by renal function group. Renal impairment had no effect on eribulin protein binding. The fraction of unbound drug was 15.2 and 18.3 % in patients with normal renal function at 30 min and 24 h postdose, respectively, and ranged from 13.4 to 18.7 % in patients with moderate and severe renal impairment for the 2 collection time points. Renal impairment decreased eribulin clearance. CL_tot_ in patients with moderate and severe renal impairment was 2.07 and 2.01 L/h, respectively, compared with 3.13 L/h in those with normal renal function. Eribulin *t*_½_ was similar in all 3 groups (43.4 h in patients with normal renal function compared with 43.9 h in those with moderate renal impairment, and 38.7 h in those with severe renal impairment).

**Table 2:** Mean (SD) pharmacokinetic parameters following single intravenous administration of eribulin in patients with normal renal function and patients with moderate or severe renal impairment on day 1

Parameter^a^	Normal renal function (*n* = 6)	Moderate renal impairment (*n* = 7)	Severe renal impairment (*n* = 6)
Eribulin mesylate dose	1.4 mg/m^2^	1.4 mg/m^2^	0.7 mg/m^2^
Dose normalized, mean (SD)			
*C*_max_, ng/mL/mg	109 (50.4)	140 (51.6)	236 (176)
AUC_0–inf_, ng h/mL/mg	408 (224)	595 (299)^a^	575 (232)
AUC_0–*t*_, ng h/mL/mg	391 (227)	532 (245)	546 (203)
Actual values, mean (SD)			
*C*_max_, ng/mL	242 (82.7)	306 (95.3)	254 (176)
AUC_0–inf_, ng h/mL	912 (434)	1320 (779)^a^	624 (230)
AUC_0–*t*_, ng h/mL	868 (433)	1200 (621)	593 (199)
*t*_½_, h	43.4 (15.3)	43.9 (10.9)^a^	38.7 (12.5)
CL_tot_, L/h	3.13 (1.65)	2.07 (1.03)^a^	2.01 (0.875)
*V*_ss_, L	144 (73.7)	86.5 (32.7)^a^	66.6 (26.8)
Fraction unbound, %, mean (SD)			
30 min postdose	15.2 (4.75)	13.8 (3.50)	16.2 (3.35)
24 h postdose	18.3 (7.05)	13.4 (7.72)^a^	18.7 (11.7)

*AUC*_*0–inf*_ area under the plasma concentration–time curve from time 0 to infinity, *AUC*_*0–t*_ area under the plasma concentration–time curve from time 0 to the last measurable concentration, *CL*_*tot*_ total clearance, *C*_*max*_ maximum observed plasma concentration, *SD* standard deviation, *t*_*½*_ terminal half-life, *V*_*ss*_ steady-state volume of distribution

^a^ *n* = 6

### Pharmacokinetic statistical analysis

Renal impairment increased both the AUC_0–inf_ and *C*_max_ of eribulin. Moderate or severe renal impairment increased mean dose-normalized AUC_0–inf_ 1.49-fold (90 % CI 0.9, 2.45) compared with normal renal function. Mean dose-normalized eribulin AUC_0–inf_ was similar in patients with moderate or severe impairment (ratio estimate, 1.00 [90 % CI 0.61, 1.65]). The increase in dose-normalized *C*_max_ was 1.31-fold (90 % CI 0.84, 2.05) for moderate renal impairment and 2.02-fold (90 % CI 1.27, 3.21) for severe renal impairment compared with normal renal function. The increase in dose-normalized *C*_max_ in the severe renal impairment group was influenced by the *C*_max_ observed in one patient (612 ng/mL), which was approximately threefold higher than *C*_max_ values for the other five patients in this group (range 162–197 ng/mL).

**Fig. 2:**
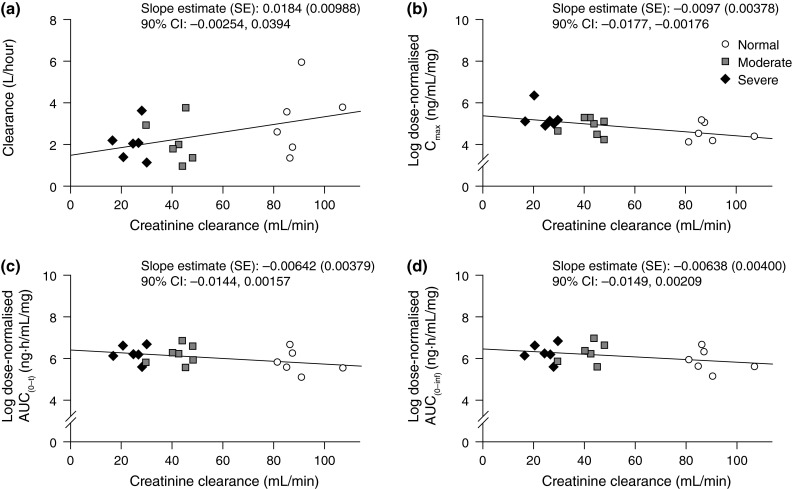
Individual eribulin plasma clearance (**a**) and log-transformed dose-normalized *C*_max_ (**b**), AUC_(0–*t*)_ (**c**) and AUC_(0–inf)_ (**d**) versus creatinine clearance. *AUC* area under the concentration–time curve, *AUC*_*0–t*_ AUC from time 0 to the last measurable concentration, *AUC*_*(0–inf)*_ area under the concentration–time curve from time zero extrapolated to infinity, *CI* confidence interval, *CL*_*tot*_ total clearance, *C*_*max*_ maximum plasma concentration, *SE* standard error

The magnitude of the slope for the linear correlation between eribulin CL_tot_ and renal function (CrCl) was small and the corresponding 90 % CI included zero (0.0184, CI −0.0025, 0.0394) (Fig. 2). The negative slopes for the linear correlations between AUC and *C*_max_ and CrCl were also numerically small and not significant (Fig. 2).

### Safety

The number of doses of eribulin mesylate received ranged from 2 to 18, with a median of 2.5, 5.0, and 5.0 doses for patients with normal renal function, moderate renal impairment, or severe renal impairment, respectively. No findings suggest any change in the established safety profile of eribulin mesylate. No patients discontinued study treatment owing to a treatment-emergent AE (TEAE) as the primary reason.

All 19 patients experienced at least 1 TEAE (defined as an AE that started after study drug administration on day 1 or re-emerged or worsened during treatment), with 17 patients (89.5 %) having AEs reported as treatment-related by the investigator. The most frequently reported TEAEs (occurring in 3 or more patients in any group) were fatigue, nausea, alopecia, decreased appetite, leukopenia and neutropenia (Table 3). TEAEs were similar across groups, with the exception of an increased incidence of alopecia, fatigue, nausea, and decreased appetite in the group with moderate renal impairment and an increased incidence of leukopenia and neutropenia in patients with normal renal function.

**Table 3:** Most frequent treatment-emergent adverse events

System organ class (preferred term)	Normal renal function (*n* = 6)	Moderate renal impairment (*n* = 7)	Severe renal impairment (*n* = 6)
Eribulin mesylate dose	1.4 mg/m^2^	1.4 mg/m^2^	0.7 mg/m^2^
TEAEs occurring in at least 3 patients in a group			
Alopecia	0	4	0
Grade 1 or 2	0	4	0
Grade 3 or 4	0	0	0
Decreased appetite	0	3	1
Grade 1 or 2	0	3	1
Grade 3 or 4	0	0	0
Fatigue	1	7	3
Grade 1 or 2	1	6	3
Grade 3 or 4	0	1	0
Leukopenia	3	1	0
Grade 1 or 2	1	0	0
Grade 3 or 4	2	1	0
Neutropenia	3	1	0
Grade 1 or 2	0	0	0
Grade 3 or 4	3	1	0
Nausea	1	4	1
Grade 1 or 2	1	4	1
Grade 3 or 4	0	0	0
TEAEs occurring in at least 2 patients in a group			
Anemia	0	2	0
Grade 1 or 2	0	2	0
Grade 3 or 4	0	0	0
Blood creatinine increased	0	2	0
Grade 1 or 2	0	2	0
Grade 3 or 4	0	0	0
Constipation	0	2	2
Grade 1 or 2	0	2	2
Grade 3 or 4	0	0	0
Dysgeusia	0	2	0
Grade 1 or 2	0	2	0
Grade 3 or 4	0	0	0
Hypertension	0	2	0
Grade 1 or 2	0	2	0
Grade 3 or 4	0	0	0
Edema, peripheral	1	0	2
Grade 1 or 2	1	0	2
Grade 3 or 4	0	0	0
Pyrexia	1	2	0
Grade 1 or 2	1	2	0
Grade 3 or 4	0	0	0
Vomiting	0	2	1
Grade 1 or 2	0	2	1
Grade 3 or 4	0	0	0

Patients with 2 or more TEAEs in the same system organ class (or with the same preferred term) are counted only once for that system organ class (or preferred term)

*TEAEs* treatment-emergent adverse events

Overall, grade 3 TEAEs occurred in five patients (26.3 %) and grade 4 TEAEs occurred in seven patients (36.8 %). Grade 3 events (highest grade) occurred in two patients with moderate renal impairment, in two patients with normal renal function, and in one patient with severe renal impairment. Grade 4 events occurred in two patients with moderate renal impairment, in one patient with severe renal impairment, and in four patients with normal renal function. There were no substantial differences among the renal groups for the incidence of grade 3 TEAEs, but owing to neutropenia and neutropenia-associated TEAEs, the incidence of grade 4 TEAEs was greater in those with normal renal function. The most frequently reported grade 3 and grade 4 TEAEs were neutropenia and neutropenia-associated TEAEs, which occurred primarily in patients with normal renal function who were administered eribulin mesylate 1.4 mg/m^2^ throughout the study.

Nine patients experienced treatment-emergent SAEs. No type (preferred term) of SAE occurred in more than one patient. SAEs reported as treatment-related occurred in two patients: dehydration and acute renal failure in one patient with severe renal impairment, and febrile neutropenia in one patient with normal renal function. One of the SAEs led to death: a patient with severe renal impairment died 31 days after a single dose of study drug, with the cause of death reported as dyspnea. The dyspnea started before study drug initiation and increased in severity to grade 2 on day 8; it was most likely related to disease progression. A second death occurred: one patient in the normal renal group died owing to progressive disease on day 27, 20 days after the last dose of study drug.

Two patients (1 in each of the moderate and severe renal impairment groups) had QTc prolongation; the first patient had at least 1 postbaseline value of >480 ms during treatment. The second patient had at least 1 postbaseline value of >480 and >500 ms. The readings were considered abnormal but not clinically significant. Both patients had baseline readings that were also considered abnormal but not clinically significant (484 and 474 ms, respectively). No clinically significant changes were observed in any other safety evaluations, including vital signs.

### Efficacy

The best overall response was stable disease, observed in ten patients. Of these, four patients were in the moderate renal impairment group (1 testicular, 1 ovarian, and 2 breast cancers), four patients were in the severe renal impairment group (1 ovarian, 1 prostate, and 2 bladder cancers), and two patients were in the normal renal function group (1 breast and 1 oral cancer). None of the patients experienced a CR or PR. Nine patients experienced progression of disease at their disease assessment.

## Discussion

The recommended starting dose of eribulin mesylate in the USA for patients with moderate renal impairment (CrCl 30–50 mL/min) is 1.1 mg/m^2^ (equivalent to eribulin 0.97 mg/m^2^, expressed as free base) administered intravenously for 2–5 min on days 1 and 8 of a 21-day cycle [4]. The current European prescribing information does not recommend any specific dose adjustments for patients with mild or moderate renal impairment, but advises that patients with severely impaired renal function (CrCl <40 mL/min) may need a dose reduction [5]. In the current study, mean eribulin exposure in patients with moderate renal impairment was 63 % higher than the mean predicted exposure in patients with normal renal function. Anticipating a greater effect of severe renal impairment on eribulin exposure, the eribulin mesylate dose was decreased to 0.7 mg/m^2^ for patients with severe renal impairment to minimize potential risks.

Only a few blood samples were collected to assess unbound eribulin concentration because eribulin has low protein binding (49–65 %) in human plasma [16]. As expected for a drug with less than 80 % protein binding, eribulin data did not show any pattern of changes in the free eribulin fraction with increasing renal impairment. Therefore, all PK assessments were performed using total eribulin concentration.

In line with previous phase I studies in patients with normal renal function, the plasma concentration–time profile of eribulin exhibited a rapid distribution phase followed by a slow elimination phase in all 3 patient groups [6, 7]. However, renal impairment did decrease eribulin clearance. Mean CL_tot_ was approximately 33 % lower in patients with moderate or severe renal impairment than in those with normal renal function. AUC and *C*_max_ were also higher in patients with moderate or severe renal impairment than in those with normal renal function. However, the dose-normalized eribulin *C*_max_ in patients with normal renal function (109 mg/mL) was lower than reported in 2 previous phase I studies (that reported ranges of 163–202 mg/mL [for doses of 0.25–1.40 mg/m^2^] [6] and 138–209 mg/mL [for doses of 0.25–4.0 mg/m^2^] [7]). In the current study, the dose-normalized *C*_max_ for patients with moderate renal impairment (140 mg/mL) was within the range previously reported in patients with normal renal function. When excluding the patient with the unusually high *C*_max_ (612 ng/mL), the *C*_max_ values for patients with severe renal impairment (162–197 ng/mL) were also within these ranges.

Given the limited urinary elimination of eribulin, the reduction in eribulin CL_tot_ is probably due to indirect effects of renal impairment on hepatic function or biliary excretion. Indeed, renal impairment may affect the nonrenal elimination of drugs that are primarily metabolised or secreted in bile by inhibiting pathways of hepatic and gut drug metabolism. The retention of waste products caused by renal impairment may impact on drug transporters such as P-glycoprotein (P-gp; of which eribulin is a substrate [17]), thereby impairing the biliary excretion of the drugs they transport. This is supported by preclinical experiments, which demonstrated that chronic renal failure in rats is associated with a decrease in intestinal P-gp protein expression and function, which could explain the increased bioavailability of drugs observed with chronic renal failure [18].

The linear correlation between the degree of renal impairment (CrCl) and eribulin clearance (CL_tot_) showed a positive trend, but the correlation was not significant and the slope was numerically small. Similarly, linear regression of CrCl and dose-normalized AUC and *C*_max_ showed negative trends with nonsignificant correlations, indicating an increase in eribulin exposure with impairment of renal function (i.e., lower CrCl). Lack of statistical significance in linear correlations between renal impairment and eribulin PK parameters reflects a relatively small effect of renal impairment on eribulin disposition, and a lack of incremental effect of severity of renal impairment on eribulin disposition (i.e., moderate and severe impairment have similar effects on eribulin AUC and *C*_max_).

Eribulin was generally well tolerated in all groups; this is consistent with data from a previous phase I trial in patients with renal dysfunction (20–40 mL/min, Cockroft–Gault, not requiring dialysis) in which eribulin was well tolerated at doses of 0.7, 1.0, and 1.4 mg/m^2^ i.v. on days 1 and 8 of a 21-day cycle [19, 20]. The toxicities experienced by patients in all groups were consistent with the known side-effect profile of eribulin. No unexpected AEs developed during treatment in patients with decreased renal function. Although the patient numbers in each group were too small to draw conclusions about specific events, there were no apparent differences in safety parameters between renal impairment groups.

**Fig. 3:**
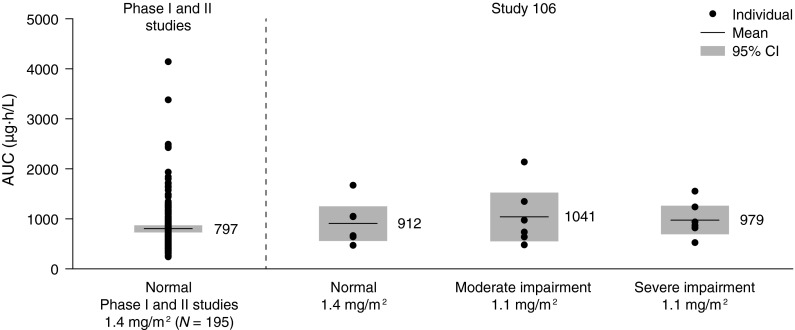
Eribulin exposure in patients with normal renal function at a dose of 1.4 mg/m^2^ and predicted eribulin exposure at a dose of 1.1 mg/m^2^ in patients with moderate or severe renal impairment. *AUC* area under the curve, *CI* confidence interval

Compared with eribulin exposure following a dose of 1.4 mg/m^2^ in patients with normal renal function, exposure to eribulin in patients with severe renal impairment following a dose of 0.7 mg/m^2^ was approximately 30 % lower, and exposure in patients with moderate impairment following a dose of 1.4 mg/m^2^ was approximately 50 % higher. Calculations were done to evaluate an intermediate dose that would achieve eribulin exposure in patients with renal impairment that was comparable to that following the recommended dose of 1.4 mg/m^2^ in patients with normal renal function. Eribulin exposure was calculated for a dose of 1.1 mg/m^2^ based on estimated CL_tot_ in patients with moderate or severe impairment. Since this study enrolled a slightly greater proportion of older females in the moderate and severe renal insufficiency groups, it is unknown if this may have contributed to reduced eribulin dosing in the setting of renal impairment. A population PK model [12] was used to predict individual exposure (AUC) in 195 patients with normal renal function dosed with eribulin mesylate 1.4 mg/m^2^ from 7 phase I studies and 1 phase II study. As shown in Fig. 3, a dose of 1.1 mg/m^2^ in patients with moderate or severe renal impairment results in a comparable eribulin exposure to that observed in patients with normal renal function dosed with eribulin mesylate 1.4 mg/m^2^ in this and previous studies. In line with this, the FDA has recently recommended to reduce the starting dose of eribulin mesylate to 1.1 mg/m^2^ in patients with moderate or severe renal impairment (CrCl 15–49 mL/min) [21].

In conclusion, renal impairment affects the PK of eribulin. Based on these findings, it is recommended that patients with moderate or severe renal impairment receive a reduced dose of 1.1 mg/m^2^ eribulin mesylate on days 1 and 8 of a 21-day cycle.
